# Acid-Sensing Ion Channels and Pain

**DOI:** 10.3390/ph3051411

**Published:** 2010-05-11

**Authors:** Qihai Gu, Lu-Yuan Lee

**Affiliations:** Department of Physiology, University of Kentucky Medical Center, 800 Rose Street, Lexington, KY 40536-0298, USA; E-Mail: lylee@uky.edu (L.Y.L.)

**Keywords:** acid sensing ion channel, acidosis, inflammation, nociceptor, pain

## Abstract

Pathophysiological conditions such as inflammation, ischemia, infection and tissue injury can all evoke pain, and each is accompanied by local acidosis. Acid sensing ion channels (ASICs) are proton-gated cation channels expressed in both central and peripheral nervous systems. Increasing evidence suggests that ASICs represent essential sensors for tissue acidosis-related pain. This review provides an update on the role of ASICs in pain sensation and discusses their therapeutic potential for pain management.

## 1. Introduction

Tissue acidosis occurs in various physiological and pathophysiological states, including inflammation, infection, ischemia, cancer, tissue injury and metabolic stress [1,2,3,4]. It has long been known that extracellular acidosis elicits pain [1,5]. The local drop in pH is first detected by primary sensory neurons. The molecular basis of proton-sensing has been actively studied since the proton-activated cation currents were observed in dorsal root ganglion (DRG) neurons almost two decades ago [6]. Increasing evidence has identified two principal types of proton-gated channels: transient receptor potential vanilloid receptor-1 (TRPV1) and acid-sensing ion channels (ASICs) [7,8]. This review provides an update on the role of ASICs in pain sensation and discusses their therapeutic potential for pain management. The involvement in pain sensation of TRPV1 and other known acid-sensitive channels such as certain two-pore domain background K^+^ channels (TASK) and purinergic P2X receptors, will not be discussed within the scope of this review. 

## 2. ASICs: An Overview

ASICs belong to the voltage-insensitive, amiloride-sensitive epithelial Na^+^ channel/degenerin (ENaC/DEG) family of cation channels [9,10]. They are widely expressed in both central and peripheral nervous systems. To date, at least four genes encoding six ASIC subunits have been cloned in mammals: ASIC1a, ASIC1b, ASIC2a, ASIC2b, ASIC3 and ASIC4 [10,11]. However, ASIC4 has not been shown to produce or modulate proton-evoked current and remains the least understood subunit [11,12]. 

### 2.1. Structure

The ASIC subunits share the same overall structure with other ENaC/DEG family members, which is characterized by two hydrophobic transmembrane domains, a large cysteine-rich extracellular loop and short intracellular N- and C-termini [10,13]. A recent study of the crystal structure of chicken ASIC1 indicates that three subunits are required to form a functional channel [14]. It is proposed that the proton sensor of ASIC protein is distributed over multiple sites, particularly His-72 and Gly-430 in the extracellular loop [13,14,15,16]. How the protein binding at these distant sites is translated into channel gating is not yet fully understood [14].

### 2.2. Property

Functional ASICs can be formed by homomultimers as well as heteromultimers [7,17]. Different homomeric and heteromeric ASIC channels have distinct kinetics, pH sensitivity, ion selectivity, tissue distribution and pharmacological properties [11,18,19,20]. The only known activator of ASICs is extracellular proton. When activated, ASICs are preferentially permeable to Na^+^, but the homomeric ASIC1a channels are also permeable to Ca^2+^ [11,21,22]. Four ASIC subunits (ASIC1a, ASIC1b, ASIC2a and ASIC3) can form functional homomultimers, whereas ASIC2b can not form functional channel by itself but may co-assemble with other ASIC subunits to form heteromultimers with new biophysical and pharmacological properties [20,23]. In heterologous cell systems, the pH of half-maximal activation (pH_0.5_) of homomeric ASIC channels differs: 6.2–6.8 for ASIC1a, 5.1–6.2 for ASIC1b, 4.1–5 for ASIC2a, and 6.2–6.7 for ASIC3 [16,17,21,24,25,26]. Most ASICs are therefore activated by changes in pH in the physiological and pathophysiological range. Both ASIC1a and ASIC1b homomeric channels generate a rapidly activating and inactivating current; ASIC2a activates and inactivates more slowly; and ASIC3 generates most rapidly activating and inactivating current but has biphasic inactivation kinetics with a sustained component [13,20]. In addition, it appears that the inactivating rate of ASIC1a, ASIC1b and ASIC2a increases with a decrease of stimulation pH (*i.e.* proton concentration increase), whereas the time constant of inactivation for ASIC3 remains constant despite changes in pH [20]. The ASIC single channel conductance has been investigated for several homomeric and heteromeric ASIC channels and ranges from 10 to 15 pS [9].

### 2.3. Distribution

Although the exact subunit composition (or subtypes) of ASICs in most neurons remains unclear, almost all ASIC subunits are known to be present in primary sensory neurons [17,18,19,27,28]. ASIC1a, ASIC1b, ASIC2b and ASIC3 are extensively expressed in small and medium nociceptive neurons [19,25,29,30]. ASIC2a and ASIC3 are also expressed in medium and large sensory neurons [19,31,32]. In the central nervous system, ASIC1a, ASIC2a and ASIC2b are widely expressed in the brain [24,33,34,35,36]. The presence of ASICs other than ASIC1a in the dorsal horn of spinal cord, where pain-related signals relay and transmitted to the brain, is less clear [37,38,39]. ASIC4, which cannot be activated by protons, has been detected in the pituitary gland, brain, spinal cord, and retina [16,40,41,42].

## 3. Role of ASICs in Pain Sensation

**Figure 1:**
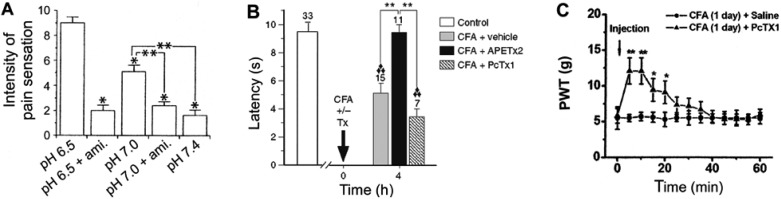
ASICs are essential detectors of acid- and inflammation-induced pain. (**A**): analgesic effect of amiloride (ami.; 200 μM), a general inhibitor of ASICs, on intradermal acid (pH 6.5 and 7.0) infusion-evoked pain in human subjects. Amiloride treatment potently suppressed the acid-evoked pain down to the pH 7.4 control level. * *P* < 0.01 *vs.* pH 6.5 treatment; ** *P* < 0.01 *vs.* pH 7.0 treatment (*n* = 10). Adapted with permission from ref. [47]. (**B**): intraplantar injection with complete Freund’s adjuvant (CFA) -induced thermal hyperalgesia in rats was prevented by APETx2 (20 μM), a specific inhibitor of ASIC3; whereas PcTx1 (120 nM), a specific inhibitor of homomeric ASIC1a, had no significant effect. Hind paw withdrawal latencies were measured at 50 °C, and the time at which inflammation was induced is indicated by the arrow. ** *P* < 0.01; ♦♦♦ *P* < 0.001, significantly different from control. Adapted with permission from ref. [50]. (**C**): CFA-induced mechanical hypersensitivity in rats, as determined by the paw withdrawal threshold (PWT) test using von Frey stimulation, is attenuated by intrathecal injection of PcTx1. * *P* < 0.05; ** *P* < 0.01 compared with intrathecal saline injection (*n* = 6). Adapted with permission from ref. [38].

Physiological pain is initiated by high-threshold unmyelinated C or myelinated Aδ primary sensory neurons that feed into noceciptive pathways of the central nervous system [43,44]. The notion that ASICs function as a major sensor of acid-evoked pain is supported by the following evidence: ASICs are expressed in peripheral sensory neurons as well as spinal nociceptive pathways (e.g., spinal cord dorsal horn); different homomeric and heteromeric ASICs are well positioned to detect and differentiate pH variations in both physiological and pathophysiological ranges; and more importantly, inhibiting ASICs has been shown to relieve pain in a variety of pain syndromes in both animals and humans ([11,23,45], Figure 1). 

### 3.1. Primary inflammatory pain

Direct perfusion of acidic solutions or iontophoresis of protons into the skin causes pain in humans [46,47,48]. This acid-evoked pain can be significantly reduced by amiloride ([47], Figure 1*A*), a common inhibitor of ASICs, and nonsteroid anti-inflammatory drugs (NSAIDs) such as declofenac and ibuprofen, which selectively inhibit ASIC1a and ASIC3, respectively [49]. Previous study shows that the human subject feels pain even at pH 7.0, which is low enough for the activation of ASIC1a and ASIC3 [46]. A recent study in rats further suggests that peripheral ASIC3 channels are the essential sensors of cutaneous acidic pain in both normal and inflammatory conditions [50]. The hypothesis was based upon these observations: first, rat cutaneous sensory neurons display a high level of ASIC3 channels, which, when activated by slight acidification (pH 7.0), depolarize the neurons and trigger action potentials; second, very moderate acidifications induce a significant increase in skin C-fibers firing, which is totally inhibited by APETx2, a specific ASIC3 inhibitor; third, primary inflammatory-induced hyperalgesia is significantly inhibited by APETx2 and also by the *in vivo* knockdown of ASIC3 with a specific siRNA, whereas PcTx1, a specific blocker of homomeric ASIC1a channels [51], has no significantly effect (Figure 1*B*).

Indeed, inflammation is known to induce a marked increase of the ASIC channel expression in primary sensory neurons; for example, the mRNA level of ASIC3 is increased by up to 15 fold in complete Freund’s adjuvant (CFA) -induced inflammation [49]. The increase in ASICs expression is largely abolished by NSAIDs in these neurons [49]. In addition, in isolated DRG neurons, a mixture of the proinflammatory mediators, including nerve growth factor, serotonin, interleukin-1 and bradykinin, increases the number of ASIC expressing neurons as well as the ASIC-like current density in these sensory neurons [52].

The involvement of ASICs in muscle and joint pain associated with inflammation has also been studied [53,54,55,56,57]. Intramuscular injection of carrageenan has been widely used to induce an animal model of chronic inflammatory muscle pain due to the local inflammation and hyperalgesia at the injection site that lasts for weeks, and can spread to the contralateral side when the inflammation becomes chronic [58]. A recent study demonstrates that, 24 hours after carrageenan-induced muscle inflammation in mice, the mRNA levels of ASIC2 and ASIC3 (but not ASIC1) in lumbar DRG neurons increase bilaterally [57]. The study also shows that both the primary and secondary hyperalgesia (increased response to noxious stimuli at and outside the site of injury, respectively) can be reversed by nonselective ASIC inhibitor A-317567. In a carrageenan-induced mouse arthritis model, the knee joint afferents with ASIC3 immunoreactivity increase from 31% to 50% after the induced joint inflammation, indicating that ASIC3 plays an important role in inflammatory joint pain [59].

The role of ASICs in primary inflammatory pain has been investigated recently in gene-targeted mice, and the data are not conclusive. In several studies mentioned above [53,54,57,59], the use of ASIC knockout mice seems to provide a clear case that ASICs, especially ASIC3, play a major role in primary inflammatory pain. However, other studies have shown that disruption of ASICs may increase the sensitivity to painful stimuli instead [60,61,62]. The reasons for these discrepancies are not known, but may be due to the variability in genetic background or species, differences in inflammatory models and research methodologies employed, complementary effect after genetic disruption, *etc.* [11,23,39,63]. 

### 3.2. Cardiac pain

Pain is the only sensation that can be evoked from most internal organs. Visceral pain can be considered as part of the defense reactions of the body against harmful stimuli, particularly of those that impinge on the mucosal lining of hollow organs [64]. It has considerable clinical relevance, and the underlying neurobiological mechanisms differ from those of somatic nociceptive or neuropathic pain [64].

Myocardial ischemia can lead to pain or discomfort within the chest, termed angina pectoris [65]. It is generally believed that such pain results from the activation of cardiac sensory neurons by a number of chemical mediators released during tissue ischemia, including lactic acid [65]. ASIC3 has been considered a major sensor for lactic acidosis created by anaerobic metabolism because it is expressed at extremely high levels in sensory neurons that innervate the heart [26,66,67]. It has been shown that both rat ASIC3 homomultimers and ASIC2a/3 heteromultimers produce sustained inward currents in response to the modest pH changes (7.3-6.7) typical of muscle ischemia; the sustained current is caused by a range of pH where there is an overlap between inactivation and activation of the ASIC channel [65]. Lactate can significantly enhance proton-evoked gating, probably mediated through a mechanism involving calcium chelation [68,69]. Studies in ASIC knockout mice further show that the acid-evoked currents from ASIC3^-/-^ cardiac afferents match the properties of ASIC2a channels, and currents from ASIC2^-/-^ cardiac afferents match the properties of ASIC3 channels [70]. These results seem to suggest that ASIC2a and ASIC3 are indeed the major ASIC subunits serving as the acid sensor in cardiac sensory neurons.

### 3.3. GI pain and respiratory sensation

Luminal acidity is a physiological challenge in the foregut, and acidosis may occur throughout the gastrointestinal (GI) tract as a result of inflammation, ischemia, cancer, microbial activity, or over distension of GI wall [71]. Acid is known to contribute to the pain associated with GI diseases such as dyspepsia, peptic ulcer, and gastro-esophageal reflux disease. ASIC1, ASIC2 and ASIC3 are all expressed in GI sensory neurons, and ASIC3 appears to be the most abundant [71]. Retrograde tracing shows that 75% of the nodose ganglion neurons and 82% of the DRG neurons projecting to the rat stomach express ASIC3 [72]. Quantitative RT-PCR of laser captured colonic neurons and fluorescence *in situ* hybridization experiments reveal that ASIC3 is the most abundant ASIC transcript within thoracolumbar DRG, followed by ASIC2 and ASIC1 [28]. In addition, the expression of ASIC3, but not ASIC1 and ASIC2, is upregulated in the colonic mucosa of patients with inflammatory bowel disease [73]. The importance of ASIC3 in modulating GI nociception is demonstrated further by studies using ASIC knockout mice. ASIC3^−/−^ mice were reported to have markedly reduced visceral mechanosensitivity when compared to control animals and ASIC1^−/−^ or ASIC2^−/−^ mice [27]. Another recent study shows that gastric acid hyperresponsiveness is absent in ASIC3^−/−^ mice but was fully preserved in ASIC2^−/−^ mice [74]. Interestingly, the non-selective ASIC channel blocker benzamil only partially attenuates mechanosensitivity in gastroesophageal afferents, whereas it markedly attenuates mechanosensitivity in colonic afferents [75]. The differential role of ASIC3 in the upper and lower GI tract indicates that this channel may serve as a key target for modulating GI nociception [76].

Most sensory inputs arising from airways and lung structures are conducted in vagus nerves and their branches, and the majority of vagal bronchopulmonary sensory nerves are nociceptive C-fibers [77,78]. Activation of bronchopulmonary C-fibers elicits irritation, cough, bronchoconstriction, burning, choking and breathless sensations [79,80]. In contrast to other visceral organs (e.g., heart, GI tract, *etc.*), pain sensation is generally not detected in the lower respiratory tract and lung parenchyma. Endogenous airway acidification has been documented in various airway inflammatory diseases including asthma and chronic obstructive pulmonary disease [2,81,82]. Airway exposure to endogenous or exogenous acid (e.g., gastroesophageal reflux with microaspiration, air pollution-induced acid fogs, *etc.*) is known to evoke cardiorespiratory symptoms such as cough, bronchospasm and dyspnea that are at least partially mediated through the activation of bronchopulmonary C-fibers and the subsequent reflex responses [82,83,84]. Recent studies have shown that physiological- and pathophysiological-relevant acidification indeed activates vagal bronchopulmonary C-fibers, which is likely mediated through the activation of both ASICs and TRPV1 [84,85]. By using retrograde labeling and multi-cell RT-PCR, a recent study shows that the mRNA of all four functional ASIC subunits (1a, 1b, 2a, and 3) are expressed in pulmonary sensory neurons [86]. Patch clamp studies show that the native ASICs expressed in these neurons are likely heteromultimers although the specific ASIC subunit combinations are not yet known [85,86]. 

### 3.4. Chronic pathological pain

Chronic pain generally falls into two categories: inflammatory and neuropathic pain [39,87]. The former is initiated by inflammation associated with tissue damage; whereas the latter is defined as “pain arising as a direct consequence of a lesion or disease affecting the somatosensory system”, and can be caused by a number of different diseases (e.g., diabetes, stroke, tumor growth, HIV infection), medical interventions (e.g., chemotherapy, surgery), and injuries (e.g., brachial plexus avulsion) [88,89,90]. It is generally believed that the exaggerated nociceptive sensation can originate from either increased sensitivity of peripheral nociceptors (peripheral sensitization), increased excitability of spinal cord dorsal horn neurons (central sensitization), or alternations in descending control from the brain [39,91,92]. Although the clinical features of inflammatory and neuropathic pain differ substantially, previous studies indicate that the local inflammation of peripheral nerves may play an important role in the generation of neuropathic pain [93,94]. In addition, these two types of chronic pain share many common central spinal and brain mechanisms [91].

ASICs play an essential role in pain sensation not only from the standpoint of their peripheral nociceptive function, but also their involvement in the development of central sensitization and pain hypersensitivity [13]. Spinal dorsal horn neurons express a high density of homomeric ASIC1a channels, and the expression of these channels is upregulated by peripheral inflammation [37,38]. Blocking of ASIC1a by spinal infusion of its specific inhibitor PcTx1 or suppression of ASIC1a expression using specific antisense oligonucleotides markedly attenuated CFA-induced thermal and mechanical hypersensitivity ([38], Figure 1*C*). In animal models of neuropathic pain, ASIC3 immunoreactivity in rat DRG neurons is elevated following lumbar disc herniation [95], and intrathecal injection of PcTx1 reverses the thermal and mechanical nociception in rats with chronic constriction injury of sciatic nerve [96]. In addition, recent studies indicate that ASIC1a may play a key role in neuronal injury after ischemic stroke [97,98,99].

## 4. ASICs as a Potential Therapeutic Target for Pain Treatment

Although extracellular proton is the only known activator of ASICs, increasing evidence shows that the proton-gating of ASICs can be modulated by a variety of factors. As we described earlier, the expression of ASICs is upregulated following inflammation and tissue injury [37,38,49,95]. Many proinflammatory mediators such as nerve growth factor, serotonin, interleukin-1 and bradykinin increase ASIC expression and acid-evoked ASIC currents [52]. The activity of ASICs, especially ASIC1a and ASIC3-containing channels, are upregulated by FMRF-amide and chronic inflammation related FMRF-amide like peptides including neuropeptide FF and neuropeptide AF [100,101,102,103,104,105]. ASICs are also modulated by a number of factors that are generated or released during ischemia, inflammation and acidosis, such as lactate [68,106], proteases [86,107,108], redox reagents [109,110], nitric oxide [111] and arachidonic acid [106,112]. In addition, many cations (such as Zn^2+^ and Ca^2+^) and protein kinases (such as PKA and PKC) can modulate certain subunit-containing ASIC channels [103]. Although the mechanisms underlying the regulatory effect of most of the ASIC modulators are not fully understood, many of them (such as lactate, proteases, nitric oxide and redox reagents) are believed to modulate the proton-dependent channel activity through an interaction with the large extracellular loop of ASIC subunits [68,107,109,111,112]. When tested in native sensory neurons or in heterogous expression systems, most ASIC subtypes are known to generate transient inward currents then desensitize rapidly [13,20]. The property of ASICs to be modulated by this large variety of endogenous chemical substances probably allows them to detect and respond to the sustained acidosis that occurs during inflammatory and many other pathological conditions [11,113]. Therefore, delineating the mechanisms underling the modulation of ASICs by these molecules might be critical for understanding the physiological and pathological roles of these channels and for establishing future targets for pharmacological intervention [113].

ASICs emerged as a potential therapeutic target for pain treatment when amiloride, a non-selective ASIC blocker, was shown to attenuate the intradermal acid infusion-evoked pain in humans [47,48]. Local injection of amiloride or its derivative benzamil significantly reduces nociceptive behaviors induced by serotonin, capsaicin or formalin under acidic conditions [114]. NSAIDs, which are known for their ability to inhibit prostaglandin synthesis and also their direct inhibition of ASICs, abolish the inflammation-induced increase in ASICs expression [49,115] and significantly reduce the acid-evoked pain [48]. Another nonselective ASIC inhibitor A-317567 can reverse both the primary and secondary hyperalgesia, and appear to be more potent than amiloride in both *in vitro* and *in vivo* preparations [57]. To date, two subunit-selective ASIC inhibitors have been identified. PcTx1, a short peptide isolated from the venom of the South American tarantula *Psalmopoeus cambridgei*, potently and specifically inhibits ASIC1a homomultimers, while does not affect ASIC1a-containing heteromultimers [51,116]. APETx2 from the sea anemone *Anthopleura elegantissima* is another ASIC blocker that selectively and effectively inhibits homo- and hetero-meric ASIC3 channels [16,117]. Given that different ASIC subtypes seem to have distinct property, tissue distribution and contribution to pain sensation, specific ASIC inhibitors can be useful as a pharmacological tool in pain research as well as a novel avenue in pain therapy [13].

## 5. Conclusions

Tissue acidosis occurs in various physiological and pathophysiological states such as inflammation, ischemia, or tissue injury. The local drop in pH is detected by primary sensory neurons, the signals of which feed into nociceptive pathways of central nervous system and produces pain sensation. Studies using ASIC-gene-targeted approaches and ASICs inhibitors in various pain models indicate that ASICs function as the essential sensors of acid-evoked pain and appear as a potential therapeutic target for pain therapy. However, a better understanding of the structure, activation and modulation mechanisms, and development of additional potent and specific ASIC inhibitors suitable for clinical use will be needed for alleviating acid-related pain syndromes.
